# Neuronal mitochondrial disaggregase CLPB ameliorates Huntington's disease pathology in mice

**DOI:** 10.7150/thno.122651

**Published:** 2026-01-01

**Authors:** Hyeonho Kim, Gaeun Hyun, Seunghye Kim, Changmo Yu, Young-gi Hong, Jihyeon Yu, Sangsu Bae, Hyun-Woo Rhee, Jaewon Ko, Ji Won Um

**Affiliations:** 1 Department of Brain Sciences, Daegu Gyeongbuk Institute of Science and Technology (DGIST), Daegu 42988, Korea.; 2 Center for Synapse Diversity and Specificity, DGIST, Daegu 42988, Korea.; 3 Department of Chemistry, Seoul National University, Seoul 08826, Korea.; 4 Genomic Medicine Institute, Seoul National University College of Medicine, Seoul 03080, Korea.; 5 Cancer Research Institute and Department of Biomedical Sciences, Seoul National University College of Medicine, Seoul 03080, Korea.

**Keywords:** Huntington's disease, ClpB, mitochondria, disaggregase, striatum, inhibitory synapse

## Abstract

**Background:** Huntington's disease (HD) is a devastating neurodegenerative disorder caused by CAG repeat expansion in the *HTT* gene, resulting in a polyglutamine-expanded huntingtin (HTT) protein that forms toxic aggregates. Although heat-shock proteins are known to facilitate the refolding or clearance of misfolded proteins, their precise role in modulating protein aggregation in HD remains unclear. Here, we explore the function of caseinolytic peptidase B (ClpB), a mitochondrial AAA+ ATPase and heat-shock protein, in maintaining proteostasis and synaptic integrity in HD.

**Methods:** We examined how CLPB loss or overexpression in human embryonic kidney 293T (HEK293T) cells impacted the aggregation of wild-type HTT (HTT-Q23) and mutant HTT (HTT-Q79). In parallel, AAV-mediated *ClpB* knockdown or overexpression was applied to the striatum of HD model mice. and HTT aggregation and inhibitory synaptic alterations were assessed. Aggregate burden was quantified via immunostaining, and inhibitory synapse density was evaluated using VGAT immunohistochemistry and electrophysiological recordings.

**Results:** In HEK293T cells, *CLPB* knockout led to abnormal aggregation of HTT-Q23 while CLPB overexpression reduced the size of HTT-Q79 aggregates. In the mouse striatum, *ClpB* knockdown increased HTT-Q23 aggregate numbers and altered HTT-Q79 aggregation morphology, whereas CLPB overexpression restored the density and size of VGAT-positive inhibitory synapses and improved inhibitory synaptic transmission in HD model mice. These effects of CLPB overexpression were associated with a reduced mitochondrial aggregation burden, suggesting that ClpB contributes to mitochondrial protein quality control.

**Conclusions:** These results demonstrate that ClpB regulates both physiological and pathological HTT aggregation and contributes to maintaining inhibitory synaptic integrity. By modulating mitochondrial proteostasis, ClpB acts as a protective factor in HD pathology, highlighting its potential as a therapeutic target for neurodegenerative disorders characterized by protein misfolding.

## Introduction

Protein aggregation is a ubiquitous hallmark of a wide range of neurodegenerative diseases, including Alzheimer's disease (AD), Huntington's disease (HD), Parkinson's disease (PD), amyotrophic lateral sclerosis (ALS), and frontotemporal dementia (FTD) 1-3. These proteinopathies are characterized by the aberrant folding, subcellular mislocalization, and accumulation of specific proteins, which ultimately leads to cellular malfunction and neuronal degeneration. To counteract aberrant protein folding and aggregation, specialized protein disaggregases have evolved as pivotal components of the protein quality control network 4, 5. A cardinal feature of aging and neurodegeneration is the collapse of proteostasis, which is marked by protein aggregate accumulation and concomitant mitochondrial dysfunction 6. While numerous neurodegenerative disease-associated proteins are linked to mitochondria 7, 8, the precise molecular mechanisms underpinning the relationship between mitochondrial dysfunction and protein aggregation remain largely obscure. Recent findings suggest that mitochondria play an active role in maintaining cellular proteostasis through pathways such as the mitochondria as guardian in cytosol (MAGIC) pathway, through which misfolded cytosolic proteins are imported into and degraded within mitochondria 9, 10. A decline in mitochondrial function under pathological conditions leads to impaired MAGIC pathway activity, compromising cytosolic proteostasis and promoting protein aggregate buildup. However, it remains to be elucidated whether MAGIC occurs in the central nervous system and, if so, which mitochondrial proteins are involved in this process.

ClpB (caseinolytic peptidase B; also known as SKD3 in humans), which is a member of the mitochondrial AAA+ domain-containing family of ATPases, functions as a molecular chaperone involved in protein quality control 11-16. Structurally, human ClpB/SKD3 comprises a globular N-terminal mitochondrial targeting signal (MTS), a short hydrophobic stretch, four ankyrin repeat (ANK) domains, a long linker helix (LH), a ring-forming nucleotide-binding domain (NBD), and a C-terminal domain (CTD) 17. ClpB is targeted to the mitochondrial intermembrane space (IMS) via its MTS and subsequently cleaved by the PARL protease to yield its mature, active disaggregase form 16. Structural studies have elucidated that dodecameric ClpB forms through the head-to-head assembly of two hexamers mediated by ANK-mediated interactions 18, 19. The ANK and NBD domains are both essential for ClpB disaggregase activity 16. For clarity, we refer to the human ortholog as CLPB and the mouse ortholog as ClpB throughout this study. Intriguingly, loss-of-function mutations in *CLPB* are associated with severe congenital neutropenia (SCN) and 3-methylglutaconic aciduria type VII (MGCA7) 20-22. Although SCN-linked *CLPB* mutations are known to be associated with greater loss of ClpB disaggregase activity compared to MGCA7-linked changes, the precise contribution of different *CLPB* mutations to the severity and expression of these disorders remains unclear. Moreover, the physiological role of ClpB in the brain and its potential association with neurological disorders remain largely unexplored.

Huntington's disease (HD) is a hereditary neurodegenerative disorder that arises from an expanded CAG trinucleotide repeat in exon 1 of the huntingtin (HTT) gene 23. Normal HTT is indispensable for embryonic development, but expansion of the CAG trinucleotide repeats in exon 1 of the Htt gene produces an elongated polyglutamine (polyQ) tract that causes adult-onset, autosomal dominant HD. HD is characterized by widespread brain atrophy and specific degeneration of the striatum, primarily affecting medium spiny neurons 24. The emerging model of HD pathogenesis highlights multiple, converging mechanisms, including transcriptional dysregulation, impaired autophagy and other protein quality control pathways, mitochondrial dysfunction, synaptic alterations, and neuroinflammation, all of which contribute to a collapse of the proteostasis network 25. Within this multifactorial framework, chronic production of mutant HTT disrupts cellular homeostasis at multiple levels, underscoring the importance of mitochondrial proteostasis as a relevant axis that is examined in the present study. Altered mitochondrial function has emerged as a significant mechanism in HD pathogenesis 24, 26, and mutant HTT has been shown to interact with translocase of the inner mitochondrial membrane 23 (TIM23) and disrupt mitochondrial function 27, 28. Mutant HTT also induces mitochondrial fragmentation and toxicity, and interferes with normal axonal transport of organelles, resulting in reduced delivery of mitochondria to synapses 29-31. Given these converging defects in proteostasis and mitochondrial integrity, we reasoned that ClpB, as an IMS-resident disaggregase, could play a unique role in buffering proteotoxic stress in HD. Unlike canonical mitochondrial foldases (e.g., HSP60, mtHSP70), ClpB possesses a disaggregase activity that enables the refolding or clearance of aggregated proteins. Our preliminary observations that mitochondrial chaperones with disaggregase activity are upregulated in HD models further supported this rationale. Together, these considerations led us to focus on ClpB as a potential modulator of HD pathogenesis.

In the present study, we first investigated whether ClpB functions as a mitochondria-localized disaggregase in neurons. We found that in the brain, ClpB is expressed as its mature form and is exclusively localized to the mitochondria. Importantly, downregulation of CLPB in heterologous cells and cultured striatal mouse neurons significantly increased the number and size of HTT aggregates, exacerbating HTT-induced toxicity and mitochondrial dysfunction. Lastly, we observed that overexpression of CLPB in a genetic HD animal model alleviated the loss of inhibitory synapse density and impaired inhibitory synaptic transmission. Collectively, our data establish ClpB as a novel mitochondrial disaggregase that is crucial for neuronal proteostasis and suggest that modulating ClpB activity represents a promising new therapeutic strategy against HD.

## Materials and Methods

**Cell culture.** HEK293T and *CLPB* knock-out (KO) HEK293T cells were cultured in Dulbecco's Modified Eagle's Medium (DMEM; WELGENE) supplemented with 10% fetal bovine serum (FBS; Tissue Culture Biologicals) and 1% penicillin-streptomycin (Thermo Fisher) at 37 °C in a humidified 5% CO_2_ atmosphere.

**Generation of CLPB knock-out HEK293T cell line.** The SpCas9 expression plasmid (p3S-Cas9HC, Addgene #43945) and sgRNA expression plasmid (pRG2, Addgene #104174) were transfected into HEK293T cells using Lipofectamine 2000 (Invitrogen, Waltham, MA, USA) according to the manufacturer's protocol. Three days post-transfection, the cells were seeded to 96-well plates and single clonal culture was performed. The CLPB target region of each clone was analyzed by targeted deep sequencing using a MiniSeq system (Illumina, San Diego, CA, USA), and the sequencing data were processed with Cas-Analyzer 32. *CLPB*-knockout clones were initially selected based on the proportion of out-of-frame mutations in the target region and subsequently confirmed by western blot analysis.

**Animals.** All animal procedures were conducted in accordance with the guidelines and protocols for rodent research (DGIST-IACUC-17122104-01) approved by the Institutional Animal Care and Use Committee at DGIST. *ClpB* knockout mice were generated at Cyagen Co., Ltd (Beijing, China) and maintained under standard temperature-controlled laboratory conditions. zQ175 Knockin (*Htt_tm1Mfc_*/190ChdiJ; Jax #027410) mice were purchased from The Jackson Laboratory. Mice were maintained on a C57BL/6J background and housed on a 12:12 light/dark schedule with unrestricted access to food and water.

**Expression constructs.** The full-length (aa 1-707) human *CLPB* cDNA (GenBank accession number: NM_030813.6) was PCR amplified and subcloned into the *Kpn*I*-Nhe*I site of the pcDNA5-Flag vector (Sigma). pcDNA5-CLPB-V5-TurboID was generated by PCR amplifying full-length human CLPB, digesting the product with *Kpn*I and *BamH*I, and subcloning the digested product into the pcDNA5-V5-TurboID vector. pcDNA5 SCO1-V5-TurboID was generated by PCR amplifying full-length human *SCO1* (GenBank accession number: NM_004589), digesting the product with *Afl*II and *Kpn*I, and subcloning the digested product into the pcDNA5-V5-TurboID vector. The shRNA lentiviral vector against mouse *ClpB* (GenBank accession number: NM_009191.4) was constructed by annealing, phosphorylating, and subcloning an oligonucleotide targeting rat/mouse ClpB (5′-GCAAGGATGCCATCTTCATCA-3′) into the *XhoI* and *XbaI* sites of a single KD vector (L-315) immediately downstream of the human H1 promoter. The lentiviral expression vector encoding shRNA-resistant full-length human CLPB was constructed by PCR amplifying the full-length region and subcloning the product into the L-313 vector 33 at the *NheI* and *BsrGI* sites. Three nucleotides (underlined) in the 5'-GCAAAGACGCAATCTTTATCA-3' sequence were then mutated to render it small hairpin RNA-resistant. The shRNA AAV against mouse ClpB was constructed by annealing, phosphorylating, and cloning an oligonucleotide targeting mouse ClpB (5′-GCAAGGATGCCATCTTCATCA-3′) into the *BamH*I and *EcoR*I sites of the pAAV-U6-mCherry vector (Cell BioLabs, Inc.). The AAV encoding full-length human ClpB was generated by PCR amplifying full-length human *CLPB*, digesting the product with *Xba*I and *BamH*I, and subcloning the product into the pAAV-T2A-tdTomato vector. Exon 1 fragments (aa 1-90 for wild-type HTT (Q23) and aa 1-146 for mutant HTT (Q79)) of the human HTT cDNA (GenBank accession number: NM_002111.8) and the full-length cDNAs encoding human SNCA (α-synuclein, aa 1-141; NM_000345.4), PRKN (Parkin, aa 1-465; NM_004562.3), TARDBP (TDP-43, aa 1-415; NM_007375.4), and mouse Mapt (Tau, aa 1-342; NM_001404015.1) were PCR-amplified and subcloned into the *Xho*I-*BamH*I site of the pEGFP-N1 vector. To generate an NLS-tagged HTT-Q79 construct, the endogenous NES sequence within pEGFP-HTT-Q79 was replaced with the nuclear localization signal (NLS) of TDP-43 (amino-acid sequence: ATLEKLMKAFESLKSF). Briefly, NLS of TDP-43 were annealed and subcloned into the *EcoRI*-*SacII* site of the pEGFP-HTT-Q79 vector. To generate an mCherry-tagged HTT-Q79 construct, the endogenous EGFP sequence within pEGFP-HTT-Q79 was replaced with that of mCherry. Briefly, mCherry sequence was PCR-amplified and subcloned into the *BamH*I site of the pEGFP-HTT-Q79 vector. pEGFP-C1-HTT-Q23 (Cat# 40261) and HTT-Q74 (Cat# 40262) were purchased from Addgene.

**Antibodies.** The following commercially available antibodies were purchased: mouse monoclonal anti-GFP (Santa Cruz); mouse monoclonal anti-FLAG (clone M2; Sigma); rabbit polyclonal anti-mCherry (Abnova); rabbit polyclonal anti-ClpB (Invitrogen); rabbit polyclonal anti-HSP60 (Invitrogen); mouse monoclonal anti-synaptophysin (clone SVP-38; Sigma); mouse monoclonal anti-β-actin (Santa Cruz); rabbit monoclonal anti-MAP2 (clone 3C19; Sigma); mouse monoclonal anti-MAP2 (clone AP-20; Sigma); mouse monoclonal anti-NeuN (Millipore); rabbit polyclonal anti-cleaved caspase3 (Cell Signaling); mouse monoclonal anti-GFAP (clone GA5; Cell Signaling); polyclonal anti-Iba-1 (WAKO); mouse monoclonal anti-DARPP-32 (clone H-3; Santa Cruz); mouse monoclonal anti-HTT (clone mEM48; Sigma); rabbit polyclonal anti-TOM20 (Santa Cruz); mouse monoclonal anti-V5 (Clone SV5-Pk1; Invitrogen), guinea pig polyclonal anti-VGLUT1 (Millipore), guinea pig polyclonal anti-VGLUT2 (Millipore), and rabbit polyclonal anti-VGAT (Synaptic Systems).

**LDH release assay.** The lactate dehydrogenase (LDH) released into culture medium was measured using a Cytotoxicity Detection Kit (Roche). The total LDH level was determined by lysing cells with 2% Triton X-100 and measuring absorbance at 490 nm using a VictorX3 Multilabel Plate Reader (PerkinElmer). Experimental values were expressed as a percentage of total LDH.

**Mitochondrial ATP quantification assay.** Mitochondrial ATP levels were measured using an ATP Assay Kit (Abcam, Cat#83355) according to the manufacturer's instructions with minor modifications. WT or *CLPB* KO HEK293T cells grown in 100 cm² dishes to ~80% confluency were collected, washed twice with PBS, detached using a cell lifter, and pelleted by centrifugation at 500 × g for 3 min at 4 °C. Cell pellets were resuspended in homogenization buffer (350 mM Tris-HCl, 250 mM NaCl, 50 mM MgCl₂, pH 7.8) containing 20 µL PMSF (100 mM) and incubated on ice for 2 min. After undergoing Dounce homogenization with an additional 200 µL of equilibrium buffer (3.5 mM Tris-HCl, 2.5 mM NaCl, 0.5 mM MgCl₂, pH 7.8), the lysates were centrifuged at 1,200 × g for 3 min to remove unbroken cells. The supernatant was then centrifuged at 15,000 × g for 2 min at 4 °C to pellet mitochondria, which were washed once with buffer A and recentrifuged under the same conditions. The obtained mitochondrial pellets were resuspended in 100 µL of assay buffer, homogenized by pipetting, and centrifuged at 13,000 × g for 5 min to remove debris. Supernatants were transferred to a 96-well plate, and 50 µL of ATP Reaction Mix (or Background Reaction Mix for controls) was added to each well. After incubation for 30 min at room temperature in the dark, absorbance was measured at 570 nm using a microplate reader, and ATP concentrations were calculated from a standard curve.

**Preparation of brain samples.** *1. Tissue fractions*. The indicated tissues were extracted from euthanized adult mice and homogenized in lysis buffer (1% Triton X-100, 50 mM Tris (pH 7.4), 150 mM NaCl, 2 mM MgCl_2_) containing a protease inhibitor cocktail (Thermo Scientific) using a Teflon/glass homogenizer. The homogenates were centrifuged at 16,000 × g for 15 min to remove unbroken cells and nuclei. *2. Mitochondrial fraction.* HEK293T cells or tissues dissected from euthanized mouse brains were homogenized, and homogenates were centrifuged at 1,000 × g for 5 min to clear unbroken cells and nuclear debris. An aliquot of the cytoplasmic fraction collected from the supernatant was then centrifuged at 10,000 × g for 10 min. The rest of the cytoplasmic fraction was layered on 15% Percoll buffer in a 1.7-ml Eppendorf tube and centrifuged at 22,000 × g for 10 min. The resulting gradient was confirmed to contain three layers. Subfractions F2 and F3 were collected into a clean Eppendorf tube and re-centrifuged at 22,000 × g for 10 min. The obtained pellet was resuspended in lysis buffer. *3. Developmental fractions*. Mouse brains were collected at various stages (E18, P1, P7, P14, P21, P28, and P100), fractionated as described above, and utilized in immunoblotting analyses.

**Proximity labeling and immunoprecipitation.** TurboID-mediated biotinylation was performed by co-transfecting cells with a CLPB-TurboID construct and the HTT-Q23-EGFP or HTT-Q79-EGFP constructs, using polyethylenimine (PEI; Polysciences). The transfected cells were incubated at 37 °C for 24-48 hours to allow for protein expression. For proximity labeling, biotin (Sigma-Aldrich) was added to the culture medium at a final concentration of 50 μM (1:1000 dilution of a 50-mM stock solution), and the mixture was incubated at 37 °C for 30 min. The cells were washed three times with Dulbecco's phosphate-buffered saline (DPBS) or HEPES buffer to remove excess biotin, and subsequently lysed in ice-cold lysis buffer containing 20 mM HEPES-NaOH (pH 7.5), 150 mM NaCl, 2 mM CaCl_2_, 2 mM MgCl_2_, and 1% Triton X-100, supplemented with protease inhibitors. The lysates were cleared by centrifugation and immunoprecipitated using an anti-GFP antibody. For detection of biotinylated proteins, membranes were incubated with streptavidin-HRP (SA-HRP; Thermo Fisher) diluted 1:10,000 in 1 × TBST for 1 hour at room temperature, and standard ECL detection was performed.

**Primary neuronal culture, transfection, immunocytochemistry, and quantitative analyses.** Cultured striatal neurons were prepared from embryonic (E18) rat brain or E17 mouse brain and immunostained, as previously described 34. The primary antibodies utilized for immunocytochemistry of transfected neurons were anti-FLAG (1:200), anti-mCherry (1:500), and anti-MAP2 (1:500). For the neuron transfection experiments, neurons at DIV8 were transfected with pcDNA5-CLPB-Flag and pEGFP-N1-HTT using a CalPhos Kit (Clontech), and immunostained at DIV14. Fluorescent image acquisition and quantitative analysis were performed according to previously published methods 34.

**Cultures of primary astrocytes and microglia.** Primary microglia and astrocytes, prepared from P0-P2 mice cerebral tissues, were cultured for 10 days, after which microglia were separated from astrocytes and oligodendrocytes by shaking for 6 hr in an orbital shaker at 180 rpm. Astrocytes were then separated from the remaining oligodendrocytes and microglia by shaking for 24 hr in an orbital shaker at 250 rpm. Complete removal of contaminating cells was ensured by repeating the shaking a second time after an interval of 1 or 2 days. Basic astrocyte growth medium consisted of DMEM/F12 (Gibco) containing 10% FBS (WELGENE), 100 units/mL penicillin, 100 mg/mL streptomycin (Gibco), and 2 mM GlutaMAX (Gibco). Upon removal, used astrocyte growth medium was sterile-filtered and stored at 4°C for up to ~1 month for use as microglia-conditioned medium. Total RNA was isolated using TRIzol Reagent (Life Technologies), and reverse transcription was performed with the ReverTra Ace qPCR RT Master Mix Kit (Toyobo). The resulting cDNA was used as template for TB Green Premix Ex Taq (Takara) reactions (20 mL volume), which were assembled in 96-well PCR plates (SPL Life science) for cycling in an CFX connect real-time system (Bio-Rad). Bio-Rad CFX manager was used for analysis of data.

**Quantitative RT-PCR.** Cultured cortical neurons and cultured mouse microglia and astrocytes were harvested for qRT-PCR. To obtain total RNA, cultured cells were processed with TRIzol reagent (Invitrogen) as recommended by the manufacturer. Cells from one well of a 12-well plate were gathered and mixed with 500 μL of TRIzol, then allowed to stand at room temperature for 5 min. After phenol-chloroform extraction, RNA was precipitated from the upper aqueous phase. cDNA was reverse transcribed from 500 ng of RNA using a ReverTra Ace-α kit (Toyobo). qPCR was performed on 1 μL of cDNA using SYBR green qPCR master mix (Takara) and a CFX96 Touch Real-Time PCR system (Bio-Rad). β-actin was amplified as an endogenous control. The sequences of the utilized primer pairs used are as follows: mouse *ClpB*, 5'-AGA GGC ACA ACA TCA CAC TAC-3' (forward) and 5'-CCG CTC TAC CTC ATG CTT AAT-3' (reverse); mouse *slitrk1*, 5'-CAC CCT ACC TGC TAA TGT ATT CC-3' (forward) and 5'-TGC TCC AAG ACC TCC TCA TA-3' (reverse); mouse *Iba-1*, 5'-TGG GAG TTA GCA AGG GAA TG-3' (forward) and 5'-CTC AGA CGC TGG TTG TCT TA-3' (reverse); and mouse *Gfap*, 5'-GCA TTT CAG CCA CAC CTT TC-3' (forward) and 5'-CCT AAT TAC ACA GAG CCA GGA C-3' (reverse).

**Generation of adeno-associated viruses, stereotaxic surgery and viral injections.** The generation of AAV vectors and the procedures for stereotaxic viral delivery were carried out according to previously published methods 35. In the present study, striatal injections were performed at the following coordinates relative to bregma: AP +1.0 mm, ML ±1.7 mm, and DV +3.5 mm.

**Electrophysiology.** Whole-cell recordings were performed in acute striatal slices using procedures described previously 35.

**Immunocytochemistry.** HEK293T cells were seeded on coverslips and transfected with plasmids encoding SCO1-V5-TurboID or CLPB-V5-TurboID. The following day, the cells were incubated with 50 μM biotin (Alfa Aesar; Cat# A14207) for 30 min at 37 °C, washed twice with DPBS, and fixed with 4% paraformaldehyde (PFA) for 15 min at room temperature. Fixed cells were permeabilized with cold methanol at -20 °C for 5 min and then blocked with 2% dBSA in PBS for 1 h at room temperature. The primary antibodies, anti-TOM20 (1: 3,000) and anti-V5 (Invitrogen; Cat# R960-25, mouse, 1:3000), were diluted in 2% dBSA in PBS and applied to the samples overnight at 4 °C. The cells were then washed, incubated with secondary antibodies [Alexa Fluor 568-conjugated anti-rabbit IgG (Invitrogen; Cat# A11011; 1:3000), Alexa Fluor 488-conjugated anti-mouse IgG (Invitrogen; Cat# R37120; 1:3000) and Alexa Fluor 647-conjugated streptavidin (Invitrogen; Cat# S21374; 1:3000)] for 1 h at room temperature. The coverslips were then washed and imaged using a confocal fluorescence microscope.

**Immunohistochemistry.** Tissue processing, sectioning, and immunohistochemical staining were carried out following previously described methods 35. Primary antibodies included VGLUT1 (1:200), VGLUT2 (1:300), VGAT (1:300), NeuN (1:500), GFAP (1:300), Iba-1 (1:500), CD68 (1:300), DARPP-32 (1:500), and mutant HTT (1:100).

**Statistics.** Data processing and statistical analyses were performed using GraphPad Prism 7.0 (RRID: SCR_002798). Unless stated otherwise, results are presented as mean ± SEM. For each experiment, a minimum of three biologically independent mice, cultures, or animal cohorts was included. Normality of the datasets was evaluated using the Shapiro-Wilk test. Depending on the distribution and experimental design, comparisons were carried out using Student's t-test or one-way ANOVA, with the Kruskal-Wallis test applied for nonparametric analyses. Post-hoc evaluations were conducted using Dunn's test, Bonferroni adjustments, t-tests, or Mann-Whitney U tests, as appropriate. The sample size (n) for each experiment is indicated in the respective figure legends and the type of replicate along with the specific statistical test used is indicated either in the main text or in the figure captions. Statistical significance was defined as p < 0.05, with exact p-values provided in the relevant figures.

## Results

### ClpB is abundantly expressed in the mitochondrial fractions of mouse brain

**Figure 1:**
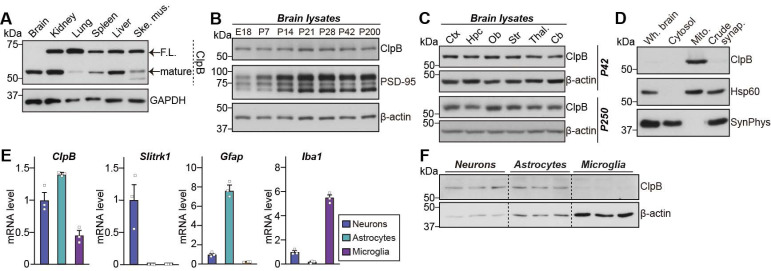
** ClpB localizes to the mitochondria in the brain. A** Tissue expression of ClpB, as revealed by immunoblot analysis with an anti-ClpB antibody. GAPDH was used as a housekeeping control. Note that ClpB appears as two bands: full-length (F.L.) and the cleaved mature form. **B** Expression levels of ClpB during development. E, embryonic day; P, postnatal day. β-actin was used for normalization. **C** Regional distribution of ClpB in various brain areas of rats at two developmental stages (P42 and P250), as determined by immunoblot analysis of brain homogenates. Cb, cerebellum; Ctx, cerebral cortex; Hpc, hippocampus; Ob, olfactory bulb; Str, striatum; Thal, thalamus. **D** Enrichment of ClpB in mitochondrial fractions. Immunoblot analysis was performed using 20 μg of protein from each fraction. Hsp60 and synaptophysin were used as markers for the mitochondrial (Mito.) and crude synaptosomal fractions (Crude synap.), respectively. **E** mRNA expression of ClpB in primary neurons, astrocytes, and microglia. *Slitrk1*, *Gfap*, and *Iba1* probes were used as markers for neurons, astrocytes, and microglia, respectively. Data are presented as means ± SEMs (n = 3 independent experiments). **F** Protein expression levels of ClpB in primary neurons, astrocytes, and microglia. β-actin was used as a housekeeping control. The uncropped blot images are provided in **Figure S6**.

To examine ClpB protein expression in brains, we first performed immunoblot analysis with different mouse tissues using anti-ClpB antibodies (**Fig. 1A**). Consistent with the previous reports that ClpB is processed by MPP and PARL 16, 36, two bands were detected: an upper band representing full-length ClpB, and a lower band corresponding to mature ClpB (**Fig. 1A**). ClpB was strongly expressed in the brain, kidney, and liver (**Fig. 1A**). Intriguingly, only the mature form of ClpB was detected in the brain, whereas both full-length and mature forms of ClpB were detected in the other tissues. ClpB protein was expressed steadily from the embryonic stage to the adult stage (**Fig. 1B**), widely distributed to a similar extent in various mouse brain areas at postnatal days 42 (P42) and P250 (**Fig. 1C**), and detected in mitochondria-enriched fractions of brain lysates (**Fig. 1D**). Quantitative RT-PCR analyses revealed that, in the brain, *ClpB* mRNA is expressed in neurons, astrocytes, and microglia (**Fig. 1E**). Consistent with this mRNA expression pattern, ClpB protein was detected in astrocytes and neurons, and to a lesser degree in microglia (**Fig. 1F**).

### CLPB deletion causes HTT aggregation and cytotoxicity in human embryonic kidney 293T cells

**Figure 2:**
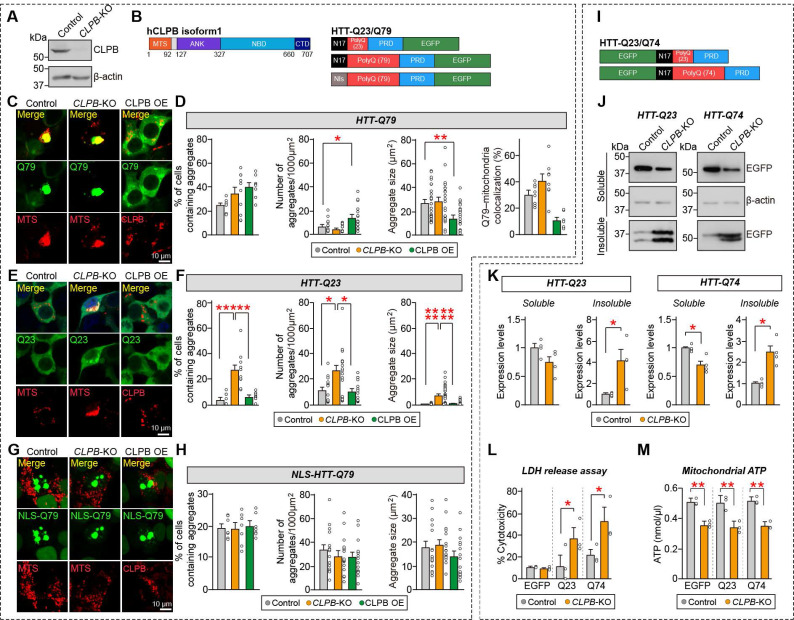
** Loss of CLPB promotes aggregation in HTT-Q23-expressing cells. A** Representative immunoblot showing CLPB expression in HEK293T wild-type (WT) and *CLPB*-knockout (KO) cells. The uncropped blot images are provided in **Figure S6**. **B** Schematics of the CLPB and HTT plasmids used in the experiments. MTS, mitochondrial targeting sequence; ANK, ankyrin repeat; NBD, nucleotide-binding domain; CTD, C-terminal domain; N17, N-terminal 17 amino acids; PRD, proline-rich domain. **C** and **E** HEK293T WT or *CLPB* KO cells were transfected with pEGFP-HTT-Q79 (**C**) or pEGFP-HTT-Q23 (**E**) together with MTS-mCherry or FLAG-tagged CLPB. Cells were subjected to immunofluorescent imaging to visualize EGFP (green), mCherry (red), or FLAG (red). Scale bars: 10 μm. **D** Quantification of HTT-Q79 aggregates: aggregate-containing cell percentage (**left**), aggregate density (**middle-left**), average aggregate size (**middle-right**), and percentage of aggregate area co-localized with mitochondria (**right**). Aggregate density (“number of aggregates/1000 μm²”) was calculated as the aggregate count normalized to the total measured cell area (based on cell boundaries shown in fluorescence images). Data are presented as means ± SEMs. Statistical significance was assessed using the Kruskal-Wallis test followed by Dunn's *post-hoc* test (*p < 0.05; **p < 0.01). **F** Quantification of HTT-Q23 aggregates: aggregate-containing cell percentage (**left**), aggregate density (**middle**), and average aggregate size (**right**). Aggregate density was calculated as described in **D**. Data are presented as means ± SEMs. Statistical significance was assessed using the Kruskal-Wallis test followed by Dunn's *post-hoc* test (*p < 0.05; **p < 0.01; ***p < 0.001; ****p < 0.0001). **G** HEK293T WT or *CLPB* KO cells were transfected with pEGFP-NLS-HTT-Q79, together with MTS-mCherry or FLAG-tagged CLPB. Cells were subjected to immunofluorescent imaging to visualize EGFP (green), mCherry (red), or FLAG (red). Scale bars: 10 μm. **H** Quantification of HTT-Q23 aggregates: aggregate-containing cell percentage (**left**), aggregate density (**middle**), and average aggregate size (**right**). Aggregate density was calculated as described in **D**. Data are presented as means ± SEMs. **I** Schematics of the HTT plasmids used in the experiments. **J** Immunoblot analysis of EGFP-HTT-Q23 and EGFP-HTT-Q74 levels in Triton X-100-soluble and -insoluble fractions. Equal volumes of each fraction were subjected to SDS-PAGE and immunoblotting for EGFP. β-actin was used as a loading control. The uncropped blot images are provided in **Figure S6**. **K** Quantification of EGFP levels in the soluble and insoluble fractions shown in **J**. Data are presented as means ± SEMs (*p < 0.05; Student t-test; n = 4 independent experiments). **L** Cytotoxicity analysis by LDH release assay in HEK293T WT and *CLPB*-KO cells transfected with pEGFP, pEGFP-HTT-Q23, or pEGFP-HTT-Q74. Data represent means ± SEMs (*p < 0.05; Student's t-test; n = 3 independent experiments). **M** Mitochondrial ATP levels in HEK293T WT and *CLPB*-KO cells transfected with pEGFP, pEGFP-HTT-Q23, or pEGFP-HTT-Q74. Data are presented as means ± SEMs (**p < 0.01; two-way ANOVA followed by Sidak's multiple comparisons test; n = 3 independent experiments).

Since ClpB was previously established as a disaggregase 16, 37, we hypothesized that neuronal ClpB might modulate various protein aggregates observed in neurodegenerative diseases. To address this hypothesis, we selected HTT protein, which has a propensity to form pathological aggregates depending on the length of the polyglutamine tract. We used the clustered regularly interspaced short palindromic repeats (CRISPR) - CRISPR-associated protein 9 (Cas9) system to generate human embryonic kidney 293T (HEK293T) cells lacking CLPB. The CRISPR-based *CLPB* knockout (KO) was assessed by immunoblot analysis, revealing that CLPB proteins were undetectable in the *CLPB*-deficient HEK293T cell lysates (**Fig. 2A**). We then transfected plasmids expressing EGFP (control), exon 1 of wild-type *HTT* gene (EGFP-tagged HTT-Q23), or exon 1 of mutant *HTT* (EGFP-tagged HTT-Q79) into wild-type (WT) or *CLPB*-deficient HEK293T cells, or co-transfected the EGFP-tagged HTT variants with the CLPB-expressing vector (**Fig. 2B**). We also co-transfected a vector encoding MTS fused with mCherry (MTS-mCherry) to visualize mitochondria in HEK293T cells. At 48 hours post-transfection, the proportion of aggregate-containing cells was evaluated by confocal microscopy. HEK293T cells expressing EGFP alone did not display any aggregates, regardless of the absence or presence of CLPB (**Fig. S1A**). Consistent with previous reports 38, 39, HTT-Q79 proteins were expressed as large aggregates inside the HEK293T cells, which also exhibited robust formation of mitochondrial aggregates that highly colocalized with HTT-Q79 (**Fig. 2C**). The percentages of cells containing aggregates in *CLPB*-KO or CLPB-overexpressing HEK293 cells were comparable to that in CLPB WT HEK293 cells (**Fig. 2D**). However, in CLPB-overexpressing cells, the HTT-Q79 aggregates were significantly smaller and there was a slight increase in the number of aggregates per cell (**Fig. 2D**). Measurement of HTT aggregation using immunocytochemical analyses failed to detect any change in aggregate size similar to that seen in the confocal microscopic analysis of *CLPB*-KO HEK293T cells (**Figs. 2C** and** 2D**), probably due to the saturation of immunofluorescent signals during the utilized imaging procedures. Quantification of the colocalization between HTT-Q79 aggregates and mitochondria revealed that, even in CLPB-overexpressing cells where aggregates appeared smaller, a substantial fraction of these aggregates remained closely associated with FLAG-CLPB-labeled mitochondria (**Figs. 2C** and** 2D**). Remarkably, *CLPB*-deficient HEK293T cells transfected with the non-aggregating HTT variants (HTT-Q23) exhibited a significant increase in the proportion of aggregate-containing cells, the number of aggregates per cell, and the aggregate size (**Figs. 2E** and** 2F**). These data indicate that *CLPB* deficiency increases the aggregation propensity of HTT-Q23, while CLPB overexpression dissociates large mutant HTT aggregates into smaller aggregates. Furthermore, when HTT-Q79 was fused with a nuclear localization signal (NLS) to restrict it to the nucleus, confocal imaging revealed that the resulting NLS-HTT-Q79 formed distinct nuclear aggregates that were completely segregated from mitochondria. In this condition, CLPB overexpression failed to reduce aggregate size or number, in contrast to its clear disaggregating effect on cytosolic HTT-Q79 (**Figs. 2G** and** 2H**). These findings indicate that CLPB's disaggregase activity requires that its substrates be able to access mitochondria.

To corroborate these results, we performed immunoblot analysis of the 1% Triton X-100-soluble and -insoluble fractions from CLPB WT or *CLPB*-KO cells expressing with either HTT-Q23 or HTT-Q74 (**Figs. 2I-K**). The levels of both HTT-Q23 and HTT-Q74 were elevated in the insoluble fraction and decreased in the soluble fraction of *CLPB*-KO cells compared to WT controls (**Figs. 2I-K**). The increased aggregation of HTT-Q23 or HTT-Q74 in *CLPB*-KO HEK293T cells was associated with increased cytotoxicity, as assessed by an LDH release assay (**Fig. 2L**). In addition, isolation of mitochondria followed by measurement of mitochondrial ATP content revealed that ATP levels were significantly reduced in *CLPB*-KO cells compared with WT controls, irrespective of whether HTT-Q23 or HTT-Q79 was expressed (**Fig. 2M**). This suggests that loss of CLPB impairs mitochondrial bioenergetic capacity independent of HTT aggregation.

We next examined whether ClpB could facilitate the aggregation of proteins involved in the pathologies of other neurodegenerative diseases, such as AD, PD, and FTD. To this end, we transfected plasmids expressing EGFP-α-synuclein, EGFP-parkin, EGFP-Tau, or EGFP-TDP43 (as a proxy for each neurodegenerative disease) into control or *CLPB*-KO HEK293T cells, or co-transfected them along with the CLPB expression vector. As a control, we also transfected cells with EGFP alone, which was observed to remain diffuse without forming aggregates, regardless of *CLPB* knockout or overexpression (**Fig. S1A**). We found that recombinant α-synuclein and Tau underwent robust aggregation in *CLPB*-KO HEK293T cells, but not in control or CLPB-overexpressing cells (**Figs. S1B** and** S1D**). This was reflected by an increased percentage of cells with aggregates, as well as a greater number and larger size of aggregates per cell in *CLPB*-KO cells (**Figs. S1B** and** S1D**). Around 40% of HEK293T cells expressing CLPB WT exhibited Parkin aggregates and this was increased to ~60%, in *CLPB*-KO HEK293T cells; however, the size of Parkin aggregates was reduced by CLPB overexpression compared with that of control HEK293T cells (**Fig. S1C**). In contrast, similar to NLS-HTT-Q79, TDP-43 formed only small nuclear aggregates that were not altered by the deletion or overexpression of CLPB (**Fig. S1E**), further supporting the notion that CLPB acts predominantly on aggregation-prone substrates accessible to mitochondria. These results suggest that CLPB is likely to play a critical role in preventing the cytosolic aggregation of a subset of neurodegenerative disease-linked proteins.

**Figure 3:**
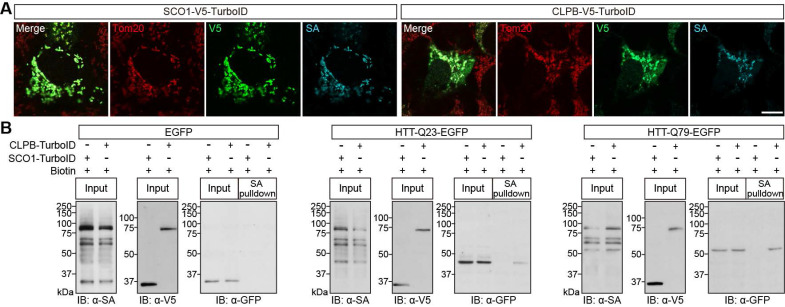
** Biotinylation of HTT via proximity labeling by mitochondrial CLPB-TurboID. A** HEK293T cells were transfected with CLPB-V5-TurboID or SCO1-V5-TurboID and subjected to immunofluorescent imaging to visualize TurboID localization (V5; green), mitochondria (Tom20; red), and biotinylated proteins (streptavidin, SA; cyan). Scale bars: 10 μm. **B** HEK293T cells were transfected with CLPB-V5-TurboID or SCO1-V5-TurboID together with EGFP alone, EGFP-HTT-Q23, or EGFP-HTT-Q79. Cell lysates were subjected to streptavidin (SA) pull-down to isolate biotinylated proteins, followed by immunoblotting with anti-GFP, anti-V5, and streptavidin-HRP to detect EGFP-tagged proteins (EGFP or EGFP-HTTs), TurboID fusion proteins, and the overall pattern of biotinylated proteins, respectively. The uncropped blot images are provided in **Figure S6**.

We next asked: How does ClpB act on these neurodegeneration-related proteins? Given that ClpB is predominantly localized in the IMS of mitochondria 40, we hypothesized that the protein aggregates enter the mitochondria and come into contact with ClpB. To test this idea, we adopted an enzyme-catalyzed proximity labeling tool 17, 37, 41. We generated CLPB-TurboID, which biotinylated the proteins within a few nanometers of CLPB when a biotin-derived small molecule substrate is added 42. As a control, we used SCO1-TurboID, a fusion of TurboID and SCO1, an IMS-localized assembly factor for the cytochrome c oxidase complex (Complex IV) 40, 43, which serves as a proximity labeling reference in the IMS. Immunofluorescence analyses revealed that the CLPB- and SCO1-TurboID constructs both co-localized with TOM20, and the streptavidin signals were predominantly localized in the mitochondrial compartment (**Fig. 3A**). To test whether CLPB-TurboID can biotinylate HTT protein aggregates, we co-expressed CLPB-TurboID with constructs encoding EGFP-tagged HTT-Q23 or HTT-Q79. Cells were treated with biotin, pull-down assays were performed using streptavidin-agarose and protein extracts, and immunoblotting analyses were undertaken using anti-EGFP antibodies. As expected, this assay detected EGFP-HTT-Q23 and EGFP-HTT-Q79, but not EGFP alone (**Fig. 3B**). These results indicate that CLPB may interact with HTT-Q23 or HTT-Q79 in mitochondria.

### ClpB deficiency aggravates HTT-induced mitochondrial fission in mouse striatal cultured neurons

**Figure 4:**
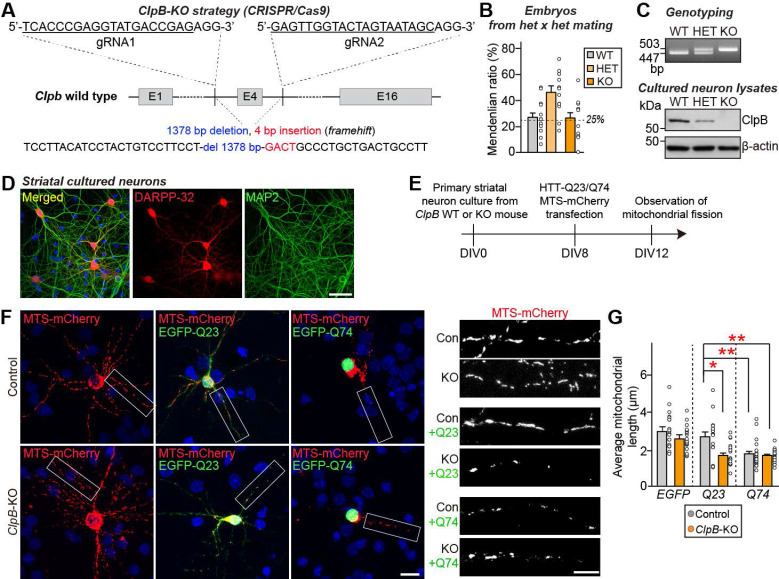
***ClpB* deletion induces mitochondrial fragmentation in HTT-Q23-expressing neurons. A** Schematic of the CRISPR-Cas9 strategy used to generate *ClpB*-KO mice. The WT locus with exons 1, 4, and 16 is depicted; exon 4 was targeted for deletion. sgRNA sequences are underlined in black and the protospacer adjacent motif (PAM) sequences are shown in red. **B** Mendelian ratio distribution of WT, heterozygous (*ClpB*^+/-^), and homozygous (*ClpB*^-/-^) embryos at embryonic day 17 (E17). **C** Genotyping results assessed by PCR (**top**) and ClpB protein expression analyzed by immunoblotting (**bottom**) in WT, *ClpB* heterozygous (Het), and *ClpB*-KO embryos or primary cultures. The uncropped blot images are provided in **Figure S6**. **D** Immunostaining of DARPP-32 (red) and MAP2 (green) in primary striatal neurons derived from E17 mouse embryos. Scale bars: 50 μm. **E** Experimental schematic showing generation of primary striatal neuron cultures from WT, *ClpB* Het, and *ClpB*-KO embryos. **F** WT and *ClpB*-KO primary striatal neurons were transfected with pEGFP-HTT-Q23 or pEGFP-HTT-Q74 along with MTS-mCherry. Cells were visualized by immunofluorescence for EGFP (green) and MTS-mCherry (red). Right panels show magnified views (white boxes) of mitochondrial morphology. **G** Quantification of average mitochondrial length in primary striatal neurons. Data are given as means ± SEMs (*p < 0.05; **p < 0.01; non-parametric Kruskal-Wallis test with Dunn's *post-hoc* test).

It is well established that the N-terminal fragment of mutant HTT is closely related to mitochondrial dysfunctions in cellular and animal models of HD 44. Thus, we next investigated the putative role of ClpB in regulating HTT aggregation and the functional significance of this modulation. To address the *in vivo* functions of ClpB, we used the CRISPR-Cas9 system to generate *ClpB*-KO mice (**Fig. 4A**). To inactivate the *ClpB* gene, sgRNAs were designed to target the* ClpB* gene upstream of exon 4 (sgRNA1) and downstream of exon 4 (sgRNA 2) (**Fig. 4A**). To assess the effects of endogenous *ClpB* deletion on HTT aggregation, we prepared primary cultured striatal neurons from mouse pups produced by crossing *ClpB* heterozygous mice. Post-genotyping from each pup revealed that embryos were produced in a normal Mendelian ratio (**Fig. 4B**). Immunoblotting analysis using anti-ClpB antibodies confirmed that ClpB levels were reduced according to the expected genotypes (**Fig. 4C**). Furthermore, immunofluorescence analysis using a medium spiny neuronal marker (dopamine- and cAMP-regulated phosphoprotein, 32 kDa; DARPP-32) revealed that the primary striatal neurons were properly prepared (**Fig. 4D**).

To investigate whether ClpB modulates the effect of HTT on mitochondrial fission, we co-transfected control and *ClpB*-KO striatal neurons with vectors expressing HTT-Q23 or HTT-Q74, together with MTS-mCherry (**Fig. 4E**), and assessed mitochondrial fragmentation by monitoring mCherry fluorescence. The exogenous expression of MTS-mCherry alone or together with HTT-Q23 was associated with the presence of filamentous mitochondrial morphology in striatal neurons, indicative of healthy neurons, and an average mitochondrial length of 2.75 μm (**Figs. 4F** and** 4G**). In line with a previous result 30, striatal neurons expressing HTT-Q74 exhibited profound mitochondrial fragmentation (**Figs. 4F** and** 4G**) and had an average mitochondrial length of 1.65 μm. There were no marked differences in HTT-Q74-induced mitochondrial fragmentation in *ClpB*-KO neurons compared to control neurons. Intriguingly, HTT-Q23-expressing *ClpB*-KO striatal neurons displayed significantly more mitochondrial fragmentation than HTT-Q23-expressing control neurons (**Figs. 4F** and** 4G**). These data suggest that ClpB is crucial for preventing mitochondrial fragmentation under physiological conditions, likely by suppressing aggregation-prone behaviors of even non-pathogenic HTT. In the absence of ClpB, HTT-Q23 tends to form aberrant aggregates that compromise mitochondrial integrity, leading to enhanced fragmentation.

### ClpB deficiency accelerates HTT aggregate formation *in vivo*

We next sought to determine whether ClpB plays a role in HTT aggregate formation *in vivo*. Because *ClpB*-KO mice exhibited early postnatal lethality (**Fig. S2A**), we decided to perform short hairpin RNA (shRNA)-mediated knockdown (KD) targeting mouse *ClpB*. Four candidate shRNAs were designed and qRT-PCR was used to assess their abilities to silence ClpB in mouse cultured cortical neurons (**Fig. S2B**). This analysis showed that only shRNA#3 was effective in this regard: It suppressed the *ClpB* mRNA level by ~ 70% (**Fig. S2B**). The efficacy of shRNA#3 against endogenous ClpB was further confirmed by semi-quantitative immunoblotting of cultured rat cortical neurons infected with lentiviruses expressing shRNA#3 or shRNA Ctrl (control), which revealed that shRNA#3 specifically decreased ClpB protein levels by ~75% in this system (**Fig. S2C**). To validate the efficiency of shRNA-mediated KD *in vivo*, the striata of adult mice were stereotactically injected with AAVs expressing shRNA#3 (sh*ClpB*) (**Figs. S3A** and **S3B**). In striata of mice injected with AAV-sh*ClpB*, ClpB protein levels were significantly reduced, but remained over 50% of the control level. This suggests that ClpB protein is substantially expressed in glia (**Figs. S3C**-**S3E**), as the AAV-sh*ClpB* viral particles were designed to transduce neurons, not non-neuronal cells (**Fig. S3F**).

**Figure 5:**
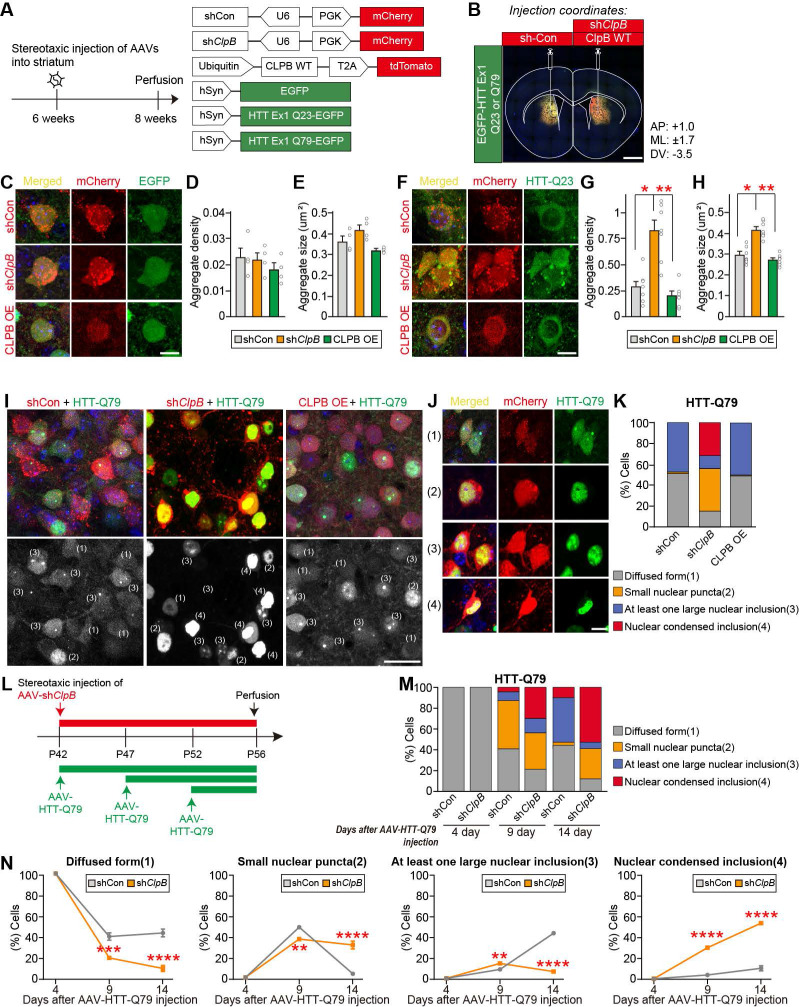
***ClpB* knockdown alters aggregation of HTT-Q23 and Q79 in the mouse striatum. A** Experimental schematic and AAV vectors expressing *ClpB* shRNAs with mCherry, CLPB WT-tdTomato, EGFP, HTT-Q23-EGFP, and HTT-Q79-EGFP. **B** Representative image of AAV injection site in the striatum. **C** Representative images showing expression of EGFP (green), mCherry (red), or tdTomato (red) following AAV injection into the striatum. Scale bar: 20 μm. **D** and **E** Quantification of aggregate density (**D**) and average aggregate size (**E**) in the striatum. Data are presented as means ± SEMs. **F** Representative images showing expression of HTT-Q23-EGFP (green), mCherry (red), or tdTomato (red) following AAV injection into the striatum. Scale bar: 20 μm. **G** and **H** Quantification of aggregate density (**G**) and average aggregate size (**H**) in the striatum. Data are presented as means ± SEMs (*p < 0.05; **p < 0.01; non-parametric Kruskal-Wallis test with Dunn's *post-hoc* test). **I** Representative images showing the expression of HTT-Q79-EGFP (green), mCherry (red), or tdTomato (red) in the striatum following AAV injection. Scale bar: 50 μm. **J** Neurons were classified into four categories based on HTT aggregate morphology: (1) diffused form, (2) small nuclear puncta, (3) at least one large nuclear inclusion, and (4) nuclear-condensed inclusion. Scale bar: 20 μm. **K** Image-based quantification of HTT aggregate morphology based on the classification criteria in (**J**). **L** Schematic illustration of the experimental design for the time-course analysis of HTT-Q79 aggregation in *ClpB*-knockdown (KD) and control mouse striata. For the 4-day and 9-day groups, AAV-sh*ClpB* was injected 14 days before analysis, and AAV-HTT-Q79 was subsequently injected 4 or 9 days before analysis, respectively. For the 14-day group, AAV-sh*ClpB* and AAV-HTT-Q79 were co-injected 14 days before analysis. **M** Stacked-bar graph summarizing the proportion of neurons exhibiting each aggregation category (diffuse, small nuclear puncta, large nuclear inclusion, and nuclear-condensed inclusion) at different time points in control and *ClpB*-KD groups. **N** Line graph showing the temporal changes in each aggregation category over time (4, 9, and 14 days) in control versus *ClpB*-KD groups. Data are presented as means ± SEMs (n = 3 per group; **p < 0.01, ***p < 0.001, ****p < 0.0001; Kruskal-Wallis test with Dunn's *post-hoc* test).

To investigate the impact of ClpB expression on HTT-related pathologies in mouse striatal neurons, we stereotactically injected mouse striata with AAV-EGFP, AAV-HTT-Q23-EGFP, or AAV-HTT-Q79-EGFP, together with AAV-sh*ClpB* or AAV-CLPB. EGFP, which was a negative control, did not exhibit any aggregation irrespective of the endogenous ClpB level (**Figs. 5A** and** 5B**). EGFP expression alone resulted in no aggregate formation, and ClpB KD or overexpression did not alter the size or density of EGFP-positive aggregates (**Figs. 5C**-**5E**), indicating that ClpB has no impact on general protein aggregation. Although HTT-Q23 did not undergo aggregation under control conditions, the number and size of aggregates containing HTT-Q23 was significantly increased in *ClpB*-KD neurons (**Figs. 5F**-**5H**). In contrast, CLPB overexpression did not affect HTT-Q23-induced aggregation (**Figs. 5F**-**5H**). These data indicate that ClpB inhibits the aggregation of HTT-Q23 in mouse striatal neurons. On the other hand, HTT-Q79 exhibited four distinct aggregation patterns in the striatal neurons 45: (1) diffuse aggregation; (2) small nuclear puncta; (3) at least one prominent nuclear inclusion; and (4) nuclear-condensed inclusions (**Figs. 5I** and** 5J**). Compared to control neurons, *ClpB*-KD neurons exhibited a marked increase in the proportions of small nuclear puncta and nuclear-condensed inclusions (**Fig. 5K**). Given that the aggregation of mutant HTT in the cytosol induces nuclear inclusion 45, our data suggest that ClpB contributes to regulating cytosolic HTT-Q79-induced aggregation, which in turn influences the nuclear inclusion of HTT aggregates. To further determine whether ClpB deficiency affects the temporal dynamics of HTT aggregation, we performed time-course analyses following AAV-mediated expression of HTT-Q79 in striatal neurons. For the 4-day and 9-day groups, AAV-shClpB was injected 14 days before analysis, and AAV-HTT-Q79 was additionally injected 4 or 9 days before analysis, respectively. For the 14-day group, AAV-shClpB and AAV-HTT-Q79 were co-injected into the striatum 14 days before analysis (**Fig. 5L**). At day 4 post-HTT-Q79 injection, both control and *ClpB*-KD neurons displayed diffuse HTT-Q79 signals, indicating that aggregation had not yet begun (**Figs. 5M** and** 5N**). By day 9, *ClpB* KD markedly altered the aggregation profile, increasing the proportion of neurons containing large or condensed nuclear inclusions while reducing diffuse and small-puncta forms (**Figs. 5M** and** 5N**). By day 14, the aggregates in both groups became more prominent, but *ClpB* KD further enhanced the fraction of condensed inclusions while diminishing intermediate aggregates, reflecting acceleration of aggregate maturation (**Figs. 5M** and** 5N**). These observations indicate that ClpB loss not only increases the aggregate burden but also accelerates the initiation and morphological progression of mutant HTT aggregation, underscoring its role in maintaining proteostasis during the early phase of HD-related pathology.

### ClpB regulates inhibitory synaptic deficits in the striatum of a mouse model of HD

**Figure 6:**
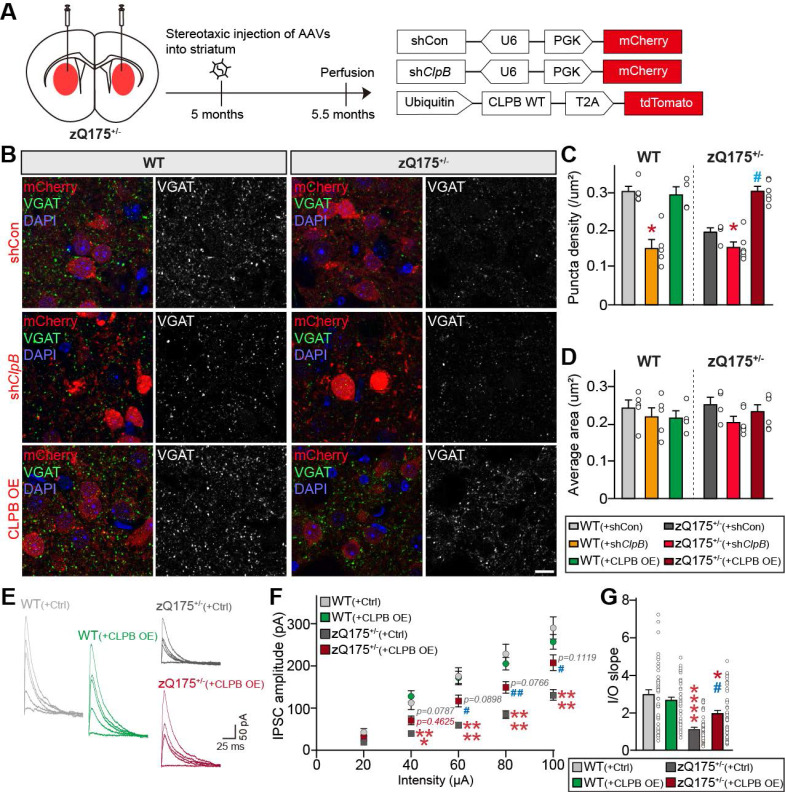
** CLPB expression restores inhibitory synaptic deficits in zQ175 mice. A** Experimental schematic and AAV constructs used for stereotactic injection. **B** Representative images of the striatum 2 weeks after injection of AAVs into WT or zQ175^+/-^ mice, immunostained for the inhibitory synapse marker, VGAT. Scale bar: 20 μm. **C** and **D** Quantification of the density (**C**) and average size (**D**) of VGAT-positive synaptic puncta. Data are presented as means ± SEMs (n=4-6 mice per group; *p < 0.05 vs. WT control; ^#^*p* < 0.05 vs. zQ175^+/-^ control; Kruskal-Wallis test with Dunn's *post hoc* test). **E** Representative evoked inhibitory postsynaptic current (eIPSCs) traces from striatal neurons. **F** Average eIPSC input-output (I-O) curve. **G** Average eIPSC I-O slope in striatal neurons from control and zQ175^+/-^ mice expressing AAV-Ctrl or AAV-CLPB. Data are presented as means ± SEMs (*p < 0.05, ***p < 0.001, ****p < 0.0001 vs. WT control; ^#^*p* < 0.05, ^##^*p* < 0.01 vs. zQ175^+/-^ control). Sample sizes were as follows: WT(+Ctrl), n = 38/9; WT(+CLPB OE), n = 43/8; zQ175^+/-^ (+Ctrl), n = 44/8; zQ175^+/-^ (+CLPB OE), n = 44/8 (n = cells/mice).

We next investigated whether ClpB regulates HTT aggregate-induced neuropathologies in an HD mouse model. We employed the widely used zQ175 knock-in (KI) mouse, which has approximately 188 CAG repeats in the *HTT* gene 46. In heterozygous zQ175 mice, mutant HTT-containing inclusions begin to accumulate around 3-12 months of age, and molecular and electrophysiological abnormalities become evident around 4-6 months 47. zQ175 mice reportedly exhibit impairments of corticostriatal and thalamostriatal excitatory synapses 48-51. To determine whether ClpB affects the synaptic deficits observed in this HD mouse model, AAVs expressing sh*Clp*B or ClpB WT were stereotactically injected into the striata of 5-month-old zQ175^+/-^ mice (**Fig. 6A**). Quantitative immunofluorescence analyses performed 2 weeks post-injection using anti-VGLUT1 and anti-VGLUT2 (to label corticostriatal and thalamostriatal projections, respectively), revealed no noticeable differences in the density or size of VGLUT1 and VGLUT2 puncta between zQ175^+/-^ and WT mice (**Fig. S4**). In addition, there were no significant differences in the development or maintenance of excitatory synapses between *ClpB*-KD and CLPB-overexpressing mice (**Fig. S4**). On the contrary, quantitative immunofluorescence analyses using anti-VGAT (vesicular GABA transporter; to label inhibitory presynapses) showed that zQ175^+/-^ mice had a significantly lower VGAT puncta density than WT mice (**Figs. 6B**-**6D**), which was in line with a previous report that GABAergic synaptic transmission was impaired in HD mouse models 52. Strikingly, the reduced VGAT puncta density was more pronounced in *ClpB*-KD mice, whereas CLPB overexpression rescued VGAT puncta density in zQ175^+/-^ mice (**Figs. 6B**-**6D**).

To corroborate these anatomical analyses, we performed whole-cell patch-clamp recordings of evoked inhibitory postsynaptic currents (eIPSCs) in striatal neurons (**Figs. 6E**-**6G**). Consistent with the reduced VGAT puncta density, eIPSC amplitudes were significantly decreased in zQ175^+/-^ mice compared to WT controls, indicating that GABAergic transmission was impaired in the HD model mice (**Figs. 6E**-**6G**). Notably, CLPB overexpression partially rescued the eIPSC amplitudes in zQ175^+/-^ mice (**Figs. 6E**-**6G**), suggesting that ClpB plays a protective role in maintaining inhibitory synaptic function in HD pathology. Together, these results support the notion that ClpB may serve as a potential therapeutic target for restoring synaptic integrity in HD.

## Discussion

Here, we elucidated the multifaceted role of ClpB, a mitochondrial AAA+ ATPase, as a critical regulator of neuronal homeostasis in models of HD. By defining its central function in maintaining cellular proteostasis and preserving mitochondrial and synaptic integrity, our findings establish ClpB as a critical component of the neuronal defense machinery against proteotoxic stress, underscoring its potential as a therapeutic target 53, 54. We provide compelling evidence that ClpB potently counteracts pathological HTT aggregation and subsequent cytotoxicity, underscoring its significance in basal proteostatic maintenance. Notably, loss of ClpB function exacerbates the aggregation propensity of even the non-pathogenic HTT-Q23 variant, further validating the fundamental importance of ClpB in basal proteostatic maintenance. Although Q23 itself is not pathogenic, the tendency of normal HTT to aggregate under ClpB deficiency highlights that ClpB appears to play a housekeeping role under physiological conditions and suggests that its loss during disease progression could exacerbate proteostasis collapse by promoting the aggregation of both normal and mutant HTT. This tendency may be explained by the fact that although wild-type HTT harbors a non-pathogenic polyQ length, it is intrinsically disordered and capable of forming transient misfolded species under basal conditions. We speculate that ClpB, potentially in collaboration with other chaperones, normally remodels or clears such intermediates, and that its loss unmasks the latent aggregation propensity of normal HTT. Such vulnerability could become pathologically relevant in aging or disease states where mitochondrial proteostasis and disaggregase activity are compromised. Although we found that ClpB protein levels did not decline in aged zQ175 mouse brains (**Fig. S5**), such stability may not necessarily translate to preserved functional capacity. In the context of chronic proteotoxic stress, the constitutive disaggregase activity of ClpB may become insufficient to counteract the progressive accumulation of misfolded HTT species, thereby contributing to proteostasis collapse despite the lack of change in ClpB expression. This is particularly compelling given that the collapse of proteostasis (a hallmark of aging) is significantly exacerbated by the presence of mutated HTT, creating a synergistic toxicity that drives HD pathogenesis 55. Further broadening the implications of the current study, we provide evidence that ClpB modulates the aggregation of other neurodegeneration-associated proteins, including α-synuclein, Tau, and Parkin, suggesting that, beyond its involvement in HD, ClpB plays a broader role in regulating proteostasis. These observations seem to indicate that there is a conserved mechanism through which ClpB functions as a core component of the mitochondrial protein quality control (mtPQC) system to play a critical role in maintaining cellular proteostasis 56. Given that mitochondrial dysfunction is a convergent hallmark in neurodegenerative disorders such as PD and AD 53, our findings suggest that ClpB-mediated mtPQC is a crucial and generalizable cellular defense process.

One of the most salient findings of the present study is that inhibitory synapses show selective susceptibility to ClpB deficiency, whereas excitatory synapses appear to be largely unaffected. In the zQ175 HD mouse model, this selective deficit is exemplified by a pronounced reduction in GABAergic synapse density without concomitant changes in excitatory synapses. This phenotype stands in marked contrast to the prevailing reports from earlier HD models 48, 50, 51, 57, in which age-dependent alterations of excitatory synaptic functions—particularly at cortico-striatal and thalamo-striatal synapses—were highlighted, often with discordant or irreconcilable conclusions. Here, 5-month-old zQ175 mice did not exhibit significant alterations in the expression of VGLUT1 or VGLUT2; however, they showed a marked reduction in VGAT expression, and this deficit was reversed by ClpB overexpression. Remarkably, KD of *ClpB* within the striatum of WT mice mirrored these inhibitory synaptic impairments, further suggesting that ClpB plays a critical role in the cell-autonomous maintenance of inhibitory synapses. The preservation of thalamostriatal and corticostriatal afferent inputs in this context provides additional support for a cell-autonomous mechanism. Our findings reveal that there is an intrinsic requirement for ClpB at inhibitory terminals, and thus complement the results of prior studies implicating non-cell-autonomous pathways with the microglia-mediated pruning of excitatory corticostriatal synapses 48. This insight helps resolve a long-standing debate regarding synapse-type-specific vulnerabilities in HD by suggesting that there is an underlying heterogeneity in the proteostatic and bioenergetic demands of different synapse types 58. We further propose that the heightened vulnerability of inhibitory synapses to expanded HTT may stem from an impairment of mitochondrial function within these specific synapses, which may fail to support the high energetic and quality-control demands of inhibitory terminals and thus contribute to the observed synaptic abnormalities. Furthermore, we document that ClpB deficiency exacerbates HTT-induced mitochondrial fragmentation, which is a key feature of the disrupted mitochondrial dynamics linked to neuronal dysfunction 59. The proper balance between mitochondrial fusion and fission is critical for neuronal health, and its disruption impairs ATP production, calcium buffering, and synaptic transmission. Thus, elucidating the mechanistic nexus connecting ClpB-mediated disaggregation, the mitochondrial fusion-fission balance, and cellular bioenergetics is essential if we hope to fully contextualize its impact on neuronal health 60.

Despite the advances made to date, the precise molecular mechanisms governing ClpB function remain incompletely understood. However, while we do not know the exact route by which cytosolic HTT accesses the mitochondrial intermembrane space, our proximity-labeling experiments performed using CLPB-TurboID place HTT within nanometer-scale proximity of CLPB. This finding strengthens the possibility that mutant HTT, or its fragments, are engaged by CLPB. This interaction may occur either following translocation into the IMS via import pathways (such as TIM23) or at specialized mitochondria-cytosol contact sites, where CLPB may sample proteins in close apposition to the outer mitochondrial membrane. Our present data confirm that mitochondrial CLPB lies in physical proximity to mutant HTT, but the relevant substrate recognition determinants and cofactor requirements are unknown. Efforts to define whether ClpB acts autonomously or in concert with other mitochondrial chaperones (e.g., HSP60) and proteases (e.g., LONP1) will be a critical next step 56. Structural biology approaches, such as cryo-EM, will be invaluable for revealing the atomic details of ClpB's disaggregase machinery. Additionally, while our *in vivo* AAV-mediated shRNA delivery-based experiments established ClpB's functional importance, the generation of conditional, neuron-specific KO, and overexpression models is needed to enable researchers to rigorously dissect the cell-autonomous roles of ClpB in disease progression. The interaction of ClpB with multiple proteins implicated in diverse neurodegenerative disorders suggests that mitochondrial proteostasis plays a common role in the etiologies of such diseases. This finding supports a conceptual shift away from therapeutic strategies focused solely on disease-specific proteins and approaches that reinforce conserved cellular defense pathways. It is important to note that the clinical manifestations of *CLPB* mutations (e.g., congenital neutropenia, cataracts, 3-methylglutaconic aciduria) are distinct from the adult-onset striatal neurodegeneration seen in HD. Our study does not propose that these disorders are mechanistically equivalent. Rather, our data support a context-specific role of ClpB, whereby germline mutations cause systemic developmental syndromes while, in HD models, ClpB activity has the capacity to buffer mutant HTT aggregation and its downstream effects. We also acknowledge that protein aggregation per se may not be the sole determinant of neurodegeneration. Intrinsically disordered proteins such as HTT can also exert pathogenic effects through altered conformational dynamics and aberrant interactions with regulatory partners. Thus, our findings should be interpreted as evidence that ClpB buffers HTT-induced proteotoxic stress within a broader context where both aggregation-dependent and -independent mechanisms contribute to disease progression. In summary, this study establishes ClpB as a critical mitochondrial disaggregase that mitigates mutant HTT aggregation, preserves inhibitory synaptic function, and maintains mitochondrial dynamics in models of HD. By linking ClpB to the broader context of aging-related proteostasis collapse and mitochondrial quality control, our work provides a mechanistic framework that holds significant implications for therapeutic strategies across a range of neurodegenerative diseases.

## Supplementary Material

Supplementary figures.

## Abbreviations

AAV: adenoassociated virus
ClpB: caseinolytic peptidase B
CRISPR: clustered regularly interspaced short palindromic repeats
eIPSC: evoked inhibitory postsynaptic current
HEK293T: human embryonic kidney 293T
HTT: Huntington's disease
MAGIC: mitochondria as guardian in cytosol
mtPQC: mitochondrial protein quality control
MTS: mitochondrial targeting signal
NLS: nuclear localization signal
VGAT: vesicular GABA transporter
VGLUT: vesicular glutamate transporter
