# Supplementary material for: Neuronal mitochondrial disaggregase CLPB ameliorates Huntington's disease pathology in mice
Source: Theranostics. 2026 Jan 1;16(5):2388–404. doi: 10.7150/thno.122651 (PMC12712926; doi:10.7150/thno.122651)
Supplement: Supplementary file 1 — Supplementary figures. [file thnov16p2388s1.pdf]

Supplementary Materials for

Neuronal Mitochondrial Disaggregase CLPB Ameliorates Huntington's Disease Pathology in Mice

Hyeonho Kim[1,2], Gaeun Hyun[1], Seunghye Kim[1,2], Changmo Yu[3], Young-gi Hong[3], Jihyeon Yu[4], Sangsu Bae[4,5], Hyun-Woo Rhee[3], Jaewon Ko[1,2,#] & Ji Won Um[1,2,#]

[1]Department of Brain Sciences, Daegu Gyeongbuk Institute of Science and Technology (DGIST), Daegu 42988, Korea
[2]Center for Synapse Diversity and Specificity, DGIST, Daegu 42988, Korea
[3]Department of Chemistry, Seoul National University, Seoul 08826, Korea
[4]Genomic Medicine Institute, Seoul National University College of Medicine, Seoul 03080, Korea
[5]Cancer Research Institute and Department of Biomedical Sciences, Seoul National University College of Medicine, Seoul 03080, Korea

[#]Correspondence: J.K. (jaewonko@dgist.ac.kr) or J.W.U. (jiwonum@dgist.ac.kr)

This file includes:
Supplementary figure S1–S6

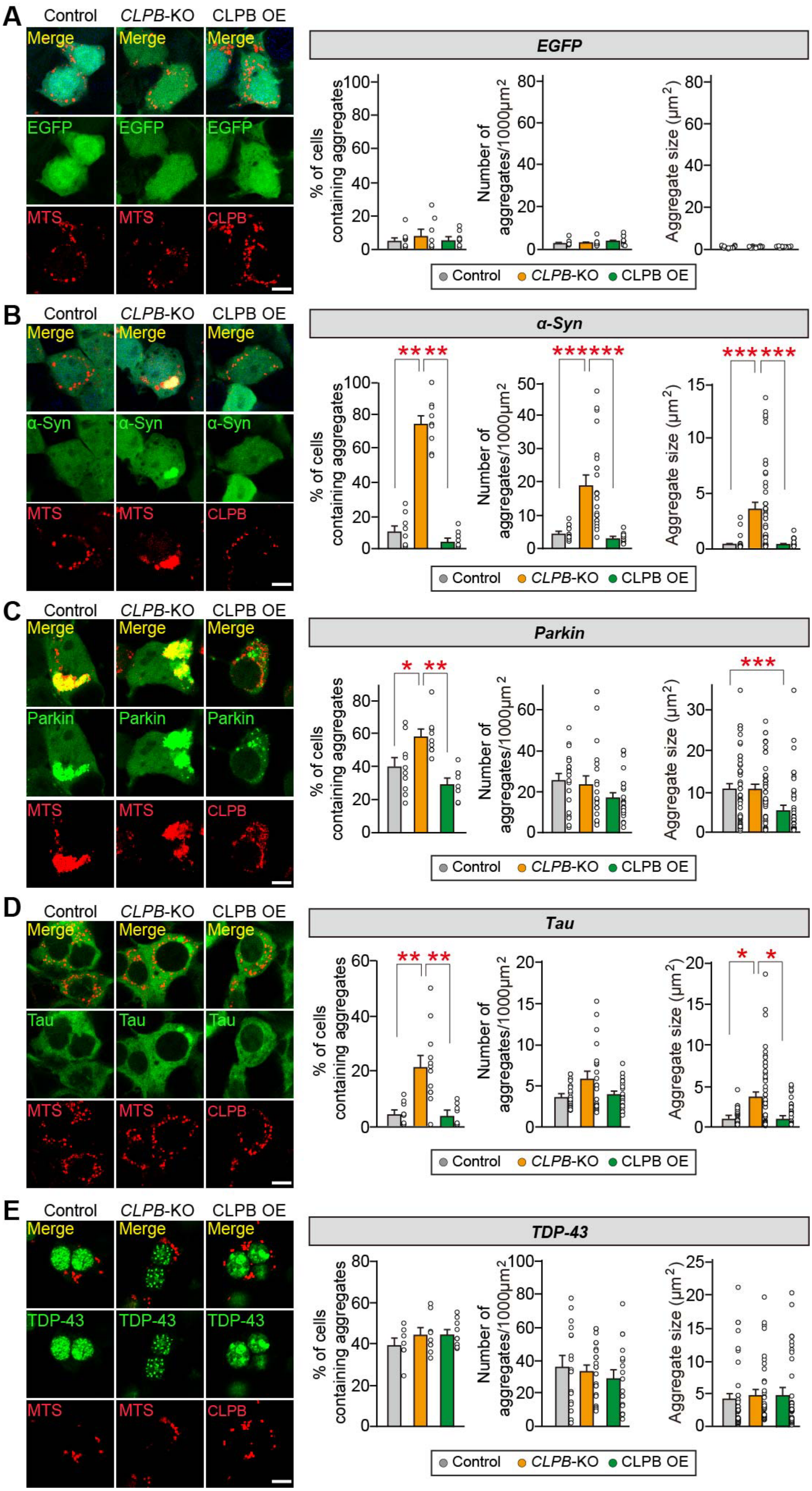
A
B
C
D
E
Control
CLPB-KO
CLPB OE
Merge
EGFP
MTS
CLPB
α-Syn
Parkin
Tau
TDP-43
% of cells containing aggregates
Number of aggregates/1000µm2
Aggregate size (µm2)
Control
CLPB-KO
CLPB OE

Supplementary Fig 1. ***CLPB*** **deletion induces aberrant aggregation of physiological forms of disease-associated proteins**

HEK293T WT or *CLPB* KO cells were transfected with EGFP alone (**A**) or EGFP-tagged α-synuclein (**B**), Parkin (**C**), Tau (**D**), or TDP-43 (**E**), together with either MTS (mitochondrial targeting sequence)-mCherry or FLAG-tagged CLPB. Cells were subjected to immunofluorescent imaging to visualize EGFP (green), mCherry (red), or FLAG (red). Scale bars: 10 μm. Quantification of the aggregate-containing cell percentage (**left**), aggregate density (**middle**), and average aggregate size (**right**). Data are presented as means ± SEMs. Statistical significance was assessed using the Kruskal–Wallis test followed by Dunn's *post-hoc* test (*$p < 0.05$; **$p < 0.01$; ***$p < 0.001$).

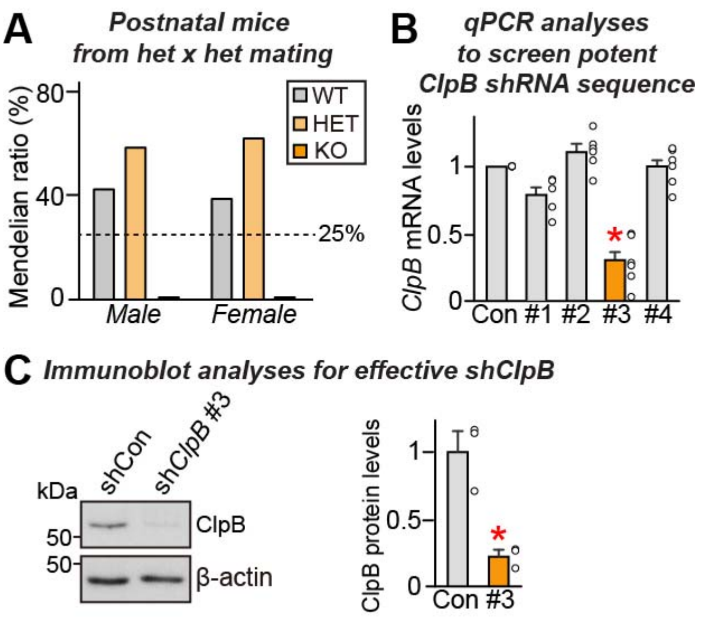

Supplementary Fig 2. Validation of a *ClpB* shRNA construct in cultured neurons
A Mendelian distribution of WT, heterozygous (*ClpB*$^{+/-}$), and homozygous (*ClpB*$^{-/-}$) postnatal mice.
B *ClpB* mRNA levels were measured by qRT-PCR in cultured cortical neurons infected at DIV3 with lentiviruses expressing four *ClpB*-targeting shRNAs (#1–#4). mRNA was prepared at DIV10. Note that shRNA#3 was the most effective at reducing *ClpB* mRNA expression.
C Cultured cortical neurons were infected at DIV3 with lentiviruses expressing *ClpB* shRNA#3, and protein lysates were collected at DIV10 for immunoblotting using the indicated antibodies. The uncropped blot images are provided in **Figure S6**. Quantification of ClpB levels normalized to the control is shown on the right.

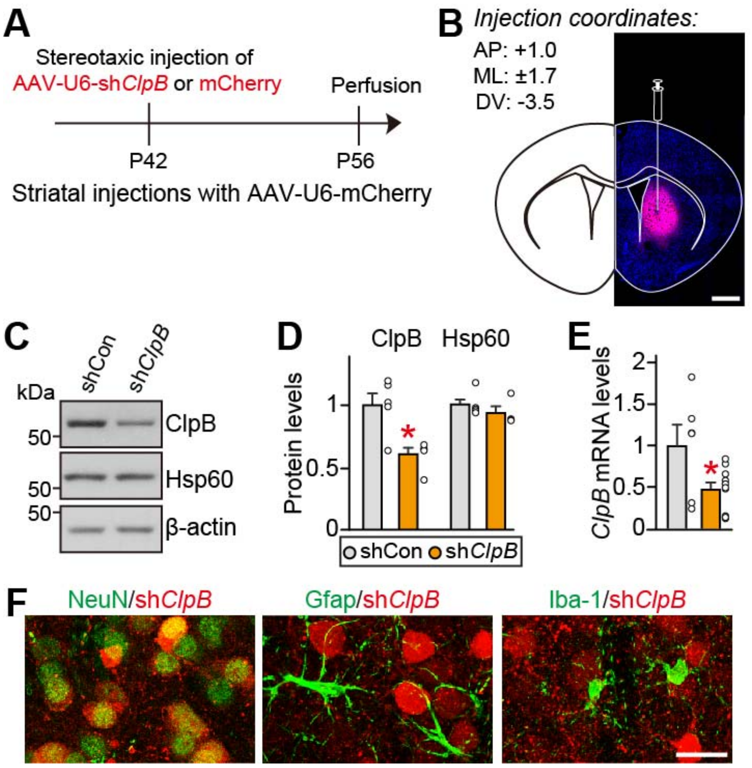

Supplementary Fig 3. Validation of a *ClpB* shRNA construct in the mouse brain

A Experimental schematics.

B Representative image showing the AAV injection site in the striatum. Scale bar: 500 μm.

C Representative immunoblot images of striatal tissues collected 2 weeks after stereotactic injection of AAV-sh*ClpB*#3 into WT mice. The uncropped blot images are provided in **Figure S6**.

D Quantification of ClpB and HSP60 protein levels from striatal tissues following AAV-sh*ClpB* injection. Data are presented as means ± SEMs from 3–5 independent experiments.

E Quantification of *ClpB* mRNA levels in striatal tissues following AAV-sh*ClpB* injection. Data are presented as means ± SEMs from 5–7 independent experiments.

F Representative images showing expression of NeuN, Gfap, Iba-1 (green), and/or sh*ClpB* (red) in the striatum following AAV-sh*ClpB* injection. Scale bar: 20 μm (applies to all images).

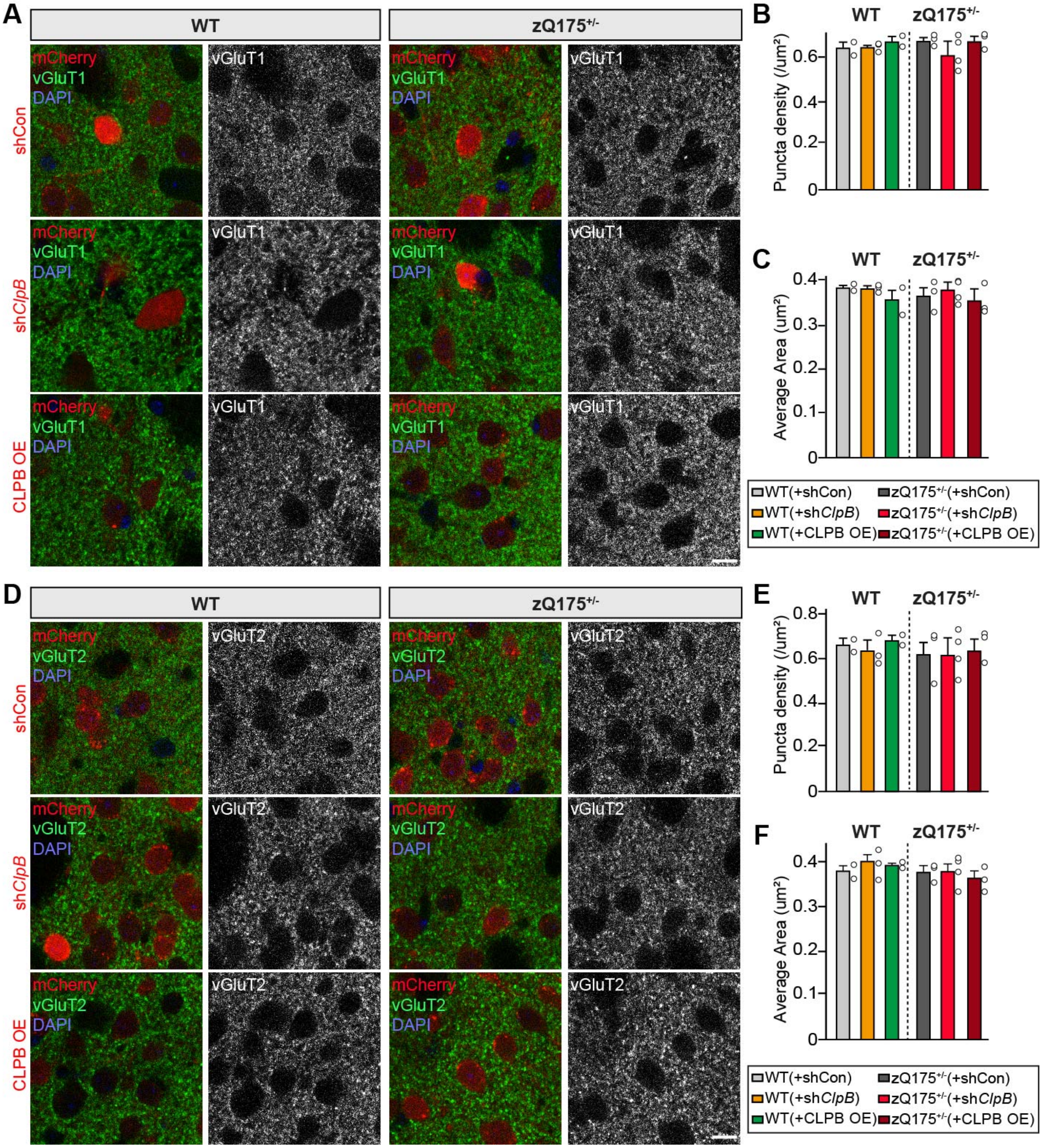

Supplementary Fig 4. zQ175 mice do not exhibit altered excitatory synaptic structure

A Representative images of the striatum 2 weeks after AAV injection into WT or zQ175$^{+/-}$ mice, immunostained for the corticostriatal excitatory synapse marker, vGluT1. Scale bar: 20 μm.

B and **C** Quantification of the density (**B**) and average size (**C**) of vGluT1^{+} synaptic puncta. Data are presented as means ± SEMs.

D Representative images of the striatum 2 weeks after AAV injection into WT or zQ175$^{+/-}$ mice, immunostained for the thalamostriatal excitatory synapse marker, vGluT2. Scale bar: 20 μm.

E and **F** Quantification of the density (**E**) and average size (**F**) of vGluT2-positive synaptic puncta. Data are presented as means ± SEMs.

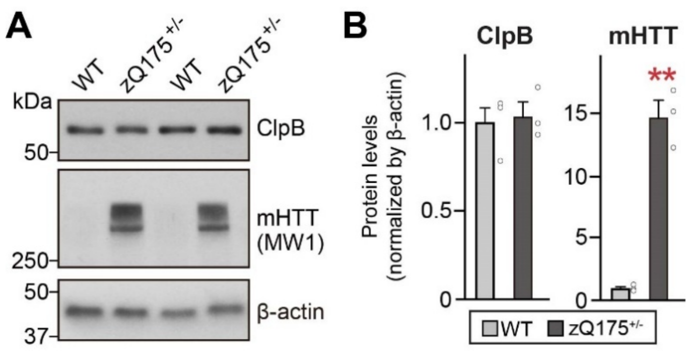

Supplementary Fig. 5. ClpB expression remains stable in aged zQ175 mice
A Representative immunoblots showing protein levels of ClpB and mutant HTT (mHTT, detected by the MW1 antibody) in striatal lysates from aged zQ175 +/– and wild-type (WT) mice. β-actin was used as a loading control. The uncropped blot images are provided in **Figure S6**.
B Quantification of ClpB and mHTT protein levels normalized to β-actin. Data are presented as means ± SEMs (n = 3 mice per group; Student's t-test).

Figure 1A

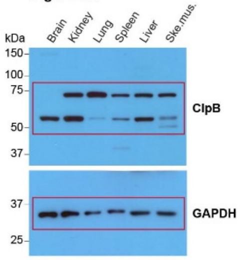

Figure 1B

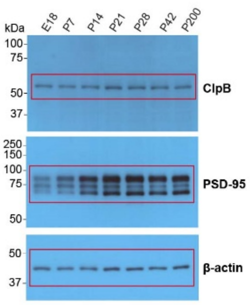

Figure 1C

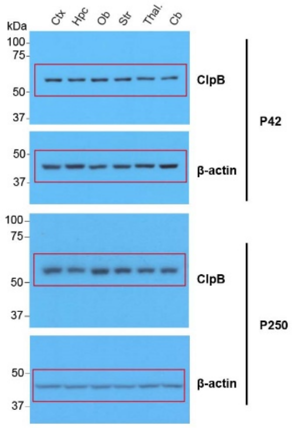

Figure 1D

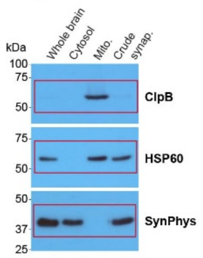

Figure 1F

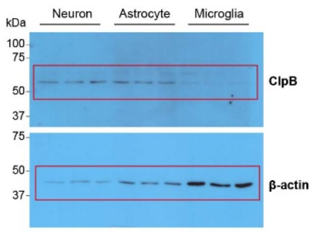

Figure 2A

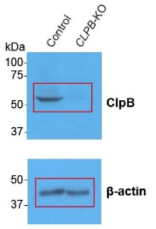

Figure 2G

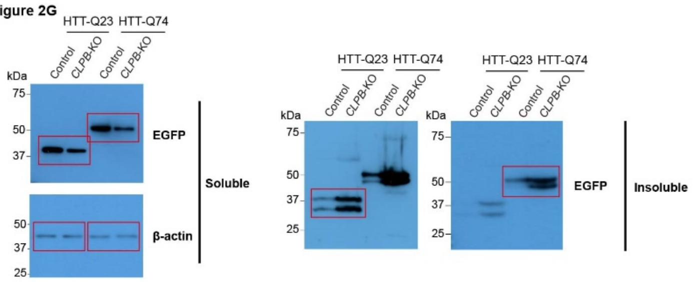

Figure 4C

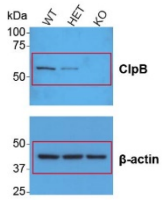

Figure 3B

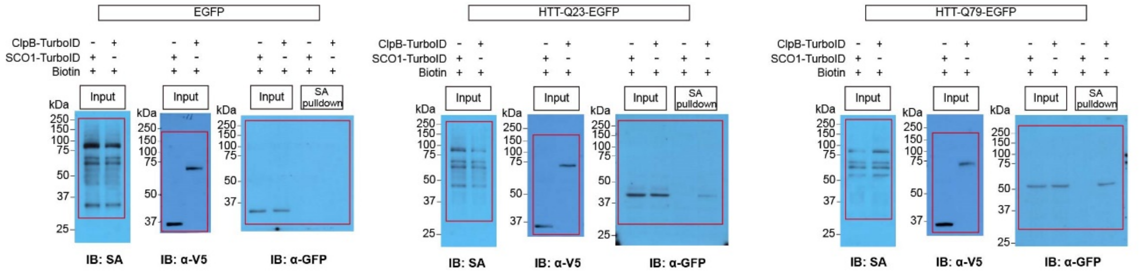

Figure S2C

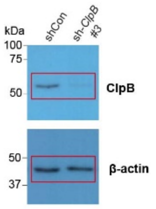

Figure S3C

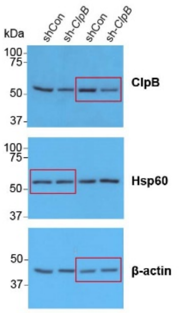

Figure S5A

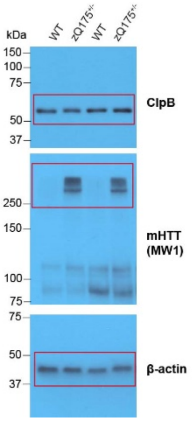

Supplementary Fig 6. Uncropped scanned images of representative immunoblots in this study
